# Enhanced performance of PTB7-Th:PCBM based active layers in ternary organic solar cells†

**DOI:** 10.1039/c8ra08919a

**Published:** 2019-03-06

**Authors:** Govinda Lakhotiya, Namdeo Belsare, Sudhir Arbuj, Bharat Kale, Abhimanyu Rana

**Affiliations:** Department of Physics, Jankidevi Bajaj College of Science Wardha 442001 India lakhotiya.govinda@gmail.com; Department of Physics, Vidyabharati Mahavidyalaya Amravati 444602 India; Centre for Materials for Electronics Technology Panchwati, Off Pashan Road Pune 411008 India; School of Engineering and Technology, BML Munjal University Gurgaon 122413 India rana.abhimanyu@gmail.com

## Abstract

The present study aims at understanding the role of incorporating Cu_2_S nanocrystals (NCs) as a third component in ternary organic solar cells. Ternary photoactive blends consisting of conjugated polymer poly[4,8-bis(5-(2-ethylhexyl)thiophen-2-yl)benzo[1,2-*b*;4,5-*b*′]dithiophene-2,6-diyl-*alt*-(4-(2-ethylhexyl)-3-fluorothieno[3,4-*b*]thiophene-)-(2-carboxylate-2-6-diyl)] (PTB7-Th), fullerene derivative phenyl-C_71_-butyric acid methyl ester (PCBM) and different wt% of Cu_2_S NCs were formulated and were employed to fabricate ternary OSCs having a device architecture of ITO/ZnO/PTB7-Th:Cu_2_S NCs:PCBM/MoO_3_/Ag. It has been observed that with the addition of 3 wt% of Cu_2_S NCs, an improved power conversion efficiency (PCE) of 8.20% is obtained against the PCE of 6.96% for reference devices. EIS measurements and AFM studies suggests that the presence of Cu_2_S NCs facilitates formation of cascading energy levels, provides smoother surfaces and helps in suppressing trap-assisted recombination.

## Abstract 2

The present study aims at understanding the role of incorporating Cu_2_S nanocrystals (NCs) as a third component in ternary organic solar cells.

## Graphical abstract

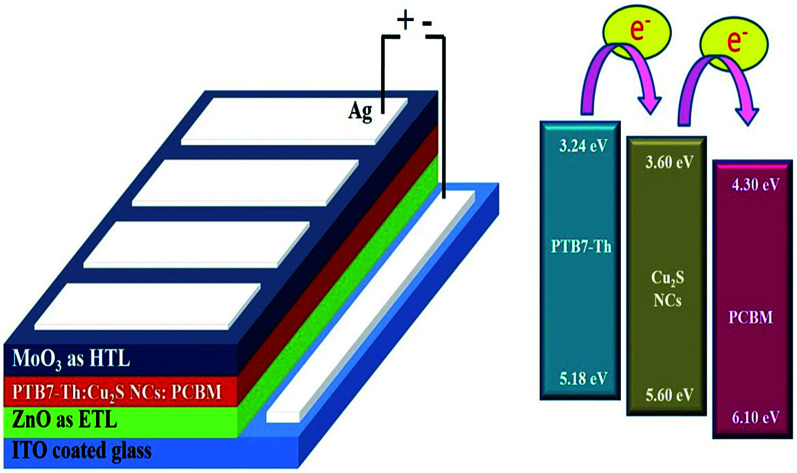

## 1. Introduction

Fabrication of bulk heterojunction ternary organic solar cells (OSCs) using ternary photoactive blends is considered as one of the recent strategies to overcome the two well-known major impediments in restricting the power conversion efficiency (PCE) of OSCs, *i.e.* narrow band gap of the donor polymer and poor mobility of carriers in both donor polymers and fullerene-based acceptors.^1–4^ It has been demonstrated that with the addition of the right third component in the binary photoactive blend, it is possible to enhance the absorption of photons, to improve the charge collection, and to do both.^4–6^ For this, as of now various materials including additional donor polymer/acceptor polymer/sensitizer (dye/metal, semiconductors and dielectric NCs)/high mobility polymers have been studied as a third component.^7–10^ Most of these materials except semiconducting NCs, are found to be capable of either enhancing the absorption of photon or improving the charge collection and not both. Some recent studies have suggested that the use of semiconducting NCs as a third component can simultaneously enhance both photon harvesting and charge collection.^11–15^ Importance of semiconducting NCs can be understood from the fact that they have tunable band-gaps which can be tuned to make it complimentary to the absorption of donor polymer and high charge carrier mobility and excellent charge transport properties which can certainly improve the charge collection.^16–18^ Further, easy way of synthesis of semiconducting NCs using solution chemistry like wet-chemical synthesis and microwave synthesis is certainly helping to realize the goal of fabricating high PCE OSCs using screen printing, inkjet printing and roll-to-roll printing, and adaptability to flexible plastic substrates.^19–23^

In the past, many metallic and semiconducting NCs have been added in ternary photoactive blends of P3HT and PCBM giving the PCE less than 5% in most of the cases.^24–32^ Some of the best known reported PCE of reference cell and best performing device upon the addition of optimum concentration of CdSe, CdS, CdTe, ZnO, Cu_2_S and FeS_2_ was found to be 3.5% and 4.2%, 0.74% and 0.95%, 0.72% and 0.79%, 2.78% and 3.39%, 1.46% and 3.39% and 2.37% and 2.89% respectively.^24–32^ Recently, Sharma *et al.*^33^ have demonstrated the use of microwave synthesized CdS nanoparticles in one of the efficient photoactive blend matrix consisting of PTB7:PCBM highlighting the increased PCE of OSCs from 6.44% to 7.14% upon the addition of optimum concentration of CdS nanoparticles. Although this is good PCE when compared to the previously reported P3HT:PCBM based ternary photoactive blend but it involves the use of toxic, hazardous and rare earth elements like cadmium and therefore can not be used for large scale fabrication.

In the present study, we have demonstrated the fabrication of high efficiency ternary OSCs by employing Cu_2_S NCs as third component in one of the well-studied photoactive blend comprised of conjugated polymer PTB7-Th (poly[4,8-bis(5-(2-ethylhexyl)thiophen-2-yl)benzo[1,2-*b*;4,5-*b*′]dithiophene-2,6-diyl-*alt*-(4-(2-ethylhexyl)-3-fluorothieno[3,4-*b*]thiophene-)-(2-carboxylate-2-6-diyl)]) and fullerene derivative PCBM (phenyl-C_71_-butyric acid methyl ester). Apparently, Cu_2_S consists of earth abundant and eco-friendly elements and also exhibit unusual properties such as high thermal and photo-chemical stability, broad absorption up to near IR range, high fluorescence quantum yields, high charge carrier mobilities and high electron affinities.^34,35^ Moreover, conduction band of Cu_2_S is located around 3.60 eV and lies between the LUMO of donor polymer PTB7-Th and fullerene acceptor PCBM.^35,36^ Thus, it is expected to form type II heterojunction with PTB7-Th and PCBM and can act as an electron cascade between them. This makes it more promising and interesting for this photoactive blend in comparison to other semiconducting NCs. However, to the best of our knowledge, there is no report on using Cu_2_S as a third component with PTB7-Th:PCBM polymers based OSCs. Here, we have studied the role of Cu_2_S NCs as third component by varying the concentration of Cu_2_S NCs in the photoactive blend of PTB7-Th:PCBM. With the inverted geometry of the devices and the addition of optimum wt% of Cu_2_S NCs, improved PCE of 8.20% is obtained as against the 6.96% for reference device. The obtained results are explained by electrochemical impedance spectroscopy (EIS) and atomic force microscopy (AFM) studies.

## 2. Experimental

### 2.1 Synthesis of Cu_2_S NCs

The synthesis of copper(i) sulfide nanocrystals (NCs) was performed using hot injection technique whose protocol was developed by Alivisatos *et al.*^34^ It deals with the chemistry of copper(ii)acetylacetonate and ammonium diethyldithiocarbamate in a mixed solvent of dodecanethiol and oleic acid whose detailed procedure can be found somewhere else.^34^ Isopropanol and toluene were used for washing to get the precipitate of required NCs and were centrifuged with 5000 rpm for 10 min.

### 2.2 Material characterization and device fabrication

For morphological studies, we have analyzed field emission scanning electron microscope (FESEM) images and high resolution transmission electron microscopy (HRTEM) images at different magnification. FESEM images were recorded using Hitachi's (S4800) field emission electron microscope which was operated at 30 kV and HRTEM images were recorded using JEOL FS2200-FEG operating at 200 kV. Cu_2_S nanocrystals (NCs) were also characterized for its phase purity by X-ray diffraction (XRD) technique using Rigaku's Miniflex 600 X-ray diffractometer which was operated at 40 kV and 30 mA using Cu K_α_ X-rays (1.54 Å). Optical property of Cu_2_S NCs was studied using UV-vis absorption spectrophotometer using Shimadzu 1800.

**Fig. 1:**
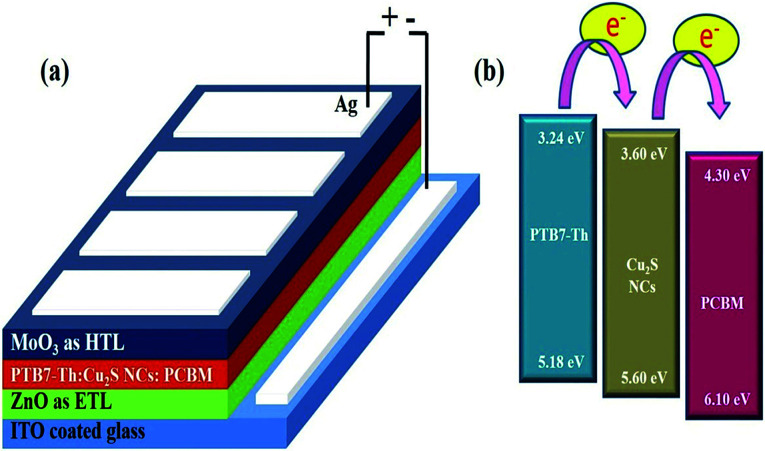
(a) Schematic highlighting device architecture ITO/ZnO/PTB7-Th:Cu_2_S NCs:PCBM/MoO_3_/Ag (b) energy levels of materials used in the solar cell device.

Devices were fabricated as per the schematic shown in the Fig. 1(a) and involves depositions of different layers. Mainly PTB7-Th, PCBM and Cu_2_S NCs based binary/ternary photoactive layer was sandwiched between the ZnO (an electron transport layer) and MoO_3_ (a hole transport layer). A pre-cleaned and patterned ITO coated glass substrates is used as a transparent substrate. Sol–gel synthesized ZnO was spin coated over ITO at 2000 rpm for 30 s and annealed at 200 °C for 15 min to obtain a film of ∼40 nm.^15^ Photoactive blend is prepared using PTB7-Th (10 mg), PCBM (15 mg) and varying amount of Cu_2_S NCs in 1 ml of chlorobenzene (CB) with 3% v/v of 1,8 diiodooctane (DIO) solvent. Ternary blend was subjected to vigorous overnight stirring at 50 °C in the dark before being spin coated over the ZnO layer.^15*a*^ For ternary photoactive blend, some amount of PCBM is replaced by the equal amount of Cu_2_S NCs. Photoactive blends were spin-coated at 1200 rpm for 60 s to obtain a film of thickness ∼100 nm and was allowed to dry for 2 h. At last, 10 nm MoO_3_ layer as a hole transport layer and 100 nm Ag electrode as an anode was deposited by thermal evaporation. Keithley 2600 source meter and a Newport solar simulator (model number 91160) with AM 1.5G spectral distribution at 1000 W m^−2^ intensity was used for *J*–*V* characteristics of the fabricated devices. Bentham's PVE300 was used for external quantum efficiency (EQE) spectra of the devices where the active device area was fixed to 7 mm^2^. Atomic force microscopy (AFM) of the films of photoactive blends with the different wt% of Cu_2_S NCs was carried out using Nanosurf's C 3000 model. The EIS studies were carried out using electrochemical work station. The frequency range was swept from 1 MHz to 1 Hz and 20 mV and AC signal was superimposed with various DC applied bias voltage.

## 3. Results and discussion

**Fig. 2:**
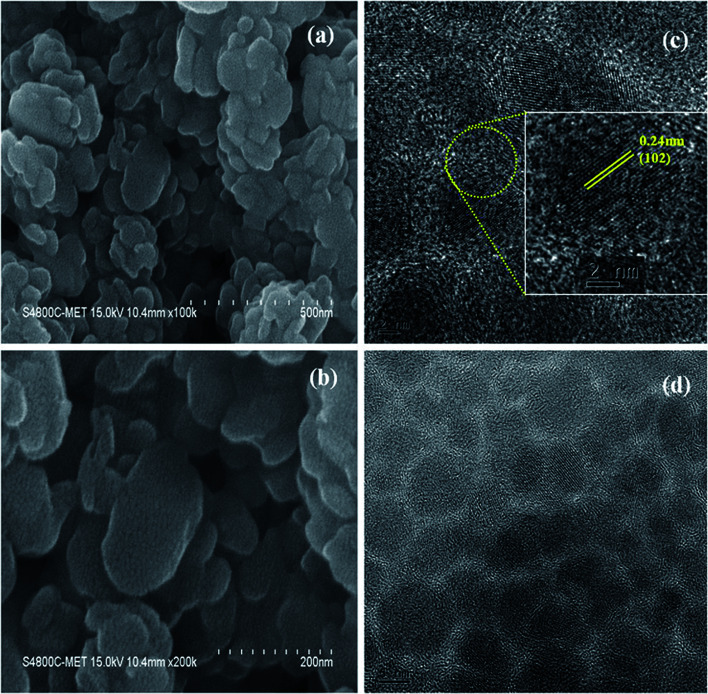
(a and b) FESEM images and (c and d) HRTEM images of as synthesized Cu_2_S NCs.

**Fig. 3:**
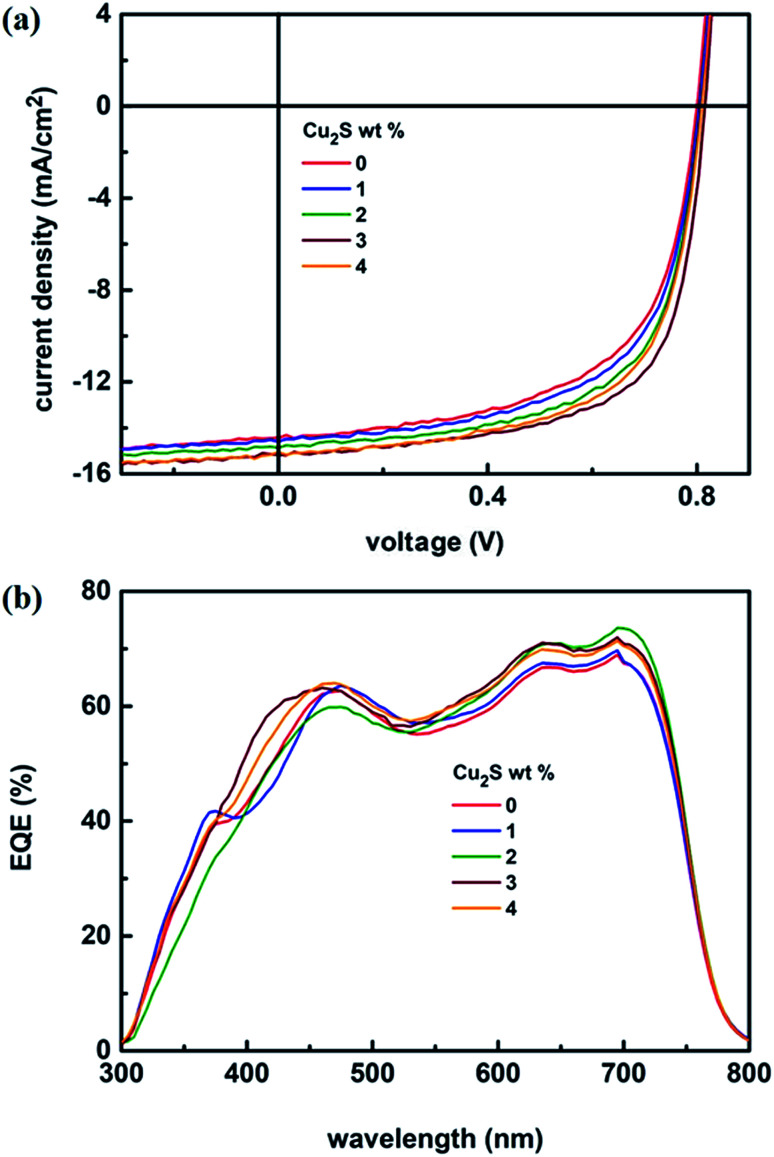
(a) *J*–*V* characteristics under illumination (AM 1.5G, one sun) and (b) EQE spectra of PTB7-Th:Cu_2_S NCs:PCBM ternary OSCs with different wt% of Cu_2_S NCs.

The schematic of full device configuration is shown in the Fig. 1(a) and the positions of conduction band and valance band of Cu_2_S NCs with respect to HOMO and LUMO levels of donor polymer PTB7-Th and acceptor polymer PCBM has been shown in Fig. 1(b). To confirm the morphology, phase purity, crystal structure and optical properties of the synthesized Cu_2_S NCs, we have recorded the FESEM and HRTEM images, XRD pattern and UV-vis absorption spectra. Fig. 2a–d shows FESEM and HRTEM images at two different magnifications. The recorded images suggest that the protocol used for the synthesis of NCs has yielded dumbbell shaped NCs having length and diameter less than 20 nm. An inter-layer spacing of 0.198 nm and 0.24 nm, as can be seen in the high magnification HRTEM image (Fig. 2(c)), corresponds to (110) and (102) planes of Cu_2_S NCs respectively. XRD pattern and UV-vis absorption spectra shown in the Fig. S1(a and b)† confirms that the as synthesized NCs are phase pure and have wide absorption spectrum UV-vis-NIR region. This is in close agreement with the previous studies.^28,34^ Current density *versus* voltage (*J*–*V*) characteristics used for the calculation of power conversion efficiency (PCE) of fabricated solar cell is shown in Fig. 3(a) and the corresponding external quantum efficiency (EQE) spectra is shown in Fig. 3(b). Various device parameters such as open circuit voltage (*V*_oc_), short-circuit current (*J*_sc_), fill factors (FF) and PCE with different wt% of Cu_2_S NCs blended in PTB7-Th:Cu_2_S NCs:PCBM ternary OSCs is summarized in Table 1.

**Table 1:** Summary of *J*–*V* characteristics of PTB7-Th:Cu_2_S NCs:PCBM devices with different wt% of Cu_2_S NCs

Cu_2_S NCs wt%	*V*_oc_ (mV)	Measured *J*_sc_ (mA cm^−2^)	Calculated *J*_sc_^a^ (mA cm^−2^)	FF (%)	PCE best^b^ (%)
0%	803	14.44	15.07	59.95	6.96 (6.85)
1%	803	14.58	14.59	61.73	7.23 (7.18)
2%	803	14.86	14.88	63.75	7.61 (7.52)
3%	818	15.17	15.20	66.10	8.20 (8.11)
4%	803	15.05	15.48	64.95	7.85 (7.76)

a Calculated from EQE data.

b Average of the five devices.

As can be seen in the Table 1, for all the wt% of Cu_2_S NCs, there is very negligible change in the *V*_oc_. To explain this, it is necessary to understand the origin of *V*_oc_. It is well known that the *V*_oc_ mainly depends on the energy difference between the HOMO of the donor polymer and the LUMO of the acceptor polymer. In the present study, for all the composition of the ternary photoactive blend, added wt% of Cu_2_S NCs is very small as compared to the donor PTB7-Th and acceptor PCBM and therefore it is most likely that at most of the interfaces within the nano-morphology of the photoactive blend, interpenetrating networks of PTB7-Th and PCBM are in direct contact with each other. As a result, the *V*_oc_ remains nearly same for all the composition. However, the scenario is different in the case of the *J*_sc_ and FF. It is observed that both *J*_sc_ and FF increases with the increase in the wt% of Cu_2_S NCs. This trend is observed till the concentration of Cu_2_S NCs reaches its optimum value of 3 wt% and fabricated ternary device has demonstrated the PCE of 8.20% as against the PCE of 6.96% for reference device. This dramatic increase in the *J*_sc_ and FF and therefore the PCE can be justified by using the theory of charge transfer complex (CTC).^37^ It might be possible that the added Cu_2_S NCs located at the interface of PTB7-Th and PCBM may get bind with PTB7-Th:PCBM *via* dipole–dipole interaction and form a charge transfer complex (CTC).^38^ This is due to the formation of the electron cascade structure arises out of the well-placed conduction band of the Cu_2_S between LUMO of donor polymer PTB7-Th and acceptor polymer PCBM. Also, it might be possible that presence of the Cu_2_S NCs in the photoactive blend provides additional energetically favorable interfaces necessary for the exciton diffusion and formation of CT states. However, when the Cu_2_S NCs concentration was further increased in the ternary photoactive blend, there is possibility that Cu_2_S NCs might have agglomerated. This agglomeration might have decreased the additional energetically favorable interfaces thereby seriously affecting the process of exciton diffusion and CT states formation. As a result, it was observed that there is a decrease in the *J*_sc_ and FF and therefore the PCE. A similar trend was observed in the case of EQE spectra. The calculated values of *J*_sc_ from EQE are shown in the Table 1. Here, it is worth mentioning that the encapsulated ternary devices were quite stable for one month without significant change in the performance. We have also fabricated the PTB7-Th:Cu_2_S NCs based binary device but found that the binary film of PTB7-Th:Cu_2_S was very rough and comprised of so many pinholes. Thus, most of the fabricated devices got short and only few of them were measurable. Few of the measureable devices yielded PCE < 1%. Therefore, this data has not been included in the manuscript. FF and *J*_sc_ is found to be highly compromised in these devices. It might be due to the poor dispersion of higher concentration of Cu_2_S NCs (15 mg ml^−1^) in PTB7-Th matrix which might have tend to agglomerate to form scattering centers.

**Fig. 4:**
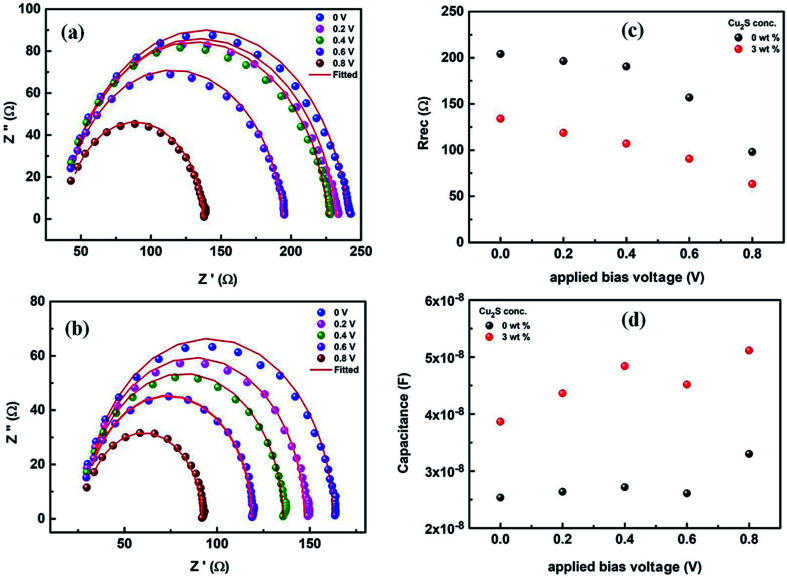
(a) and (b) Nyquist plots of PTB7-Th:Cu_2_S NCs:PCBM ternary OSCs with 0 and 3 wt% of Cu_2_S NCs respectively at different applied bias voltage (c) recombination resistance (*R*_rec_) and (d) chemical capacitance (*C*_μ_) as a function of applied bias voltage for devices with 0 and 3 wt% of Cu_2_S NCs.

To get further insight about the improved performance parameters of the ternary OSCs with optimum wt% (3%) of Cu_2_S NCs, EIS and AFM studies were carried out. Fig. 4(a) shows the Nyquist plots for both reference device (Cu_2_S NCs, 0 wt%) as well as the best performing device (Cu_2_S NCs, 3 wt%) respectively. It is observed that for both the systems, EIS spectra comprised of only one semi-circle. Therefore, they can be fitted by a simple *R*_rec_. *C*_μ_ equivalent circuit, where *R*_rec_ is a recombination resistance (*R*_rec_) and *C*_μ_ is the chemical capacitance (*C*_μ_). However, as can be noticed, in both the cases, obtained Nyquist plots are not perfect semi-circles. This truncated semi-circle arises mainly due to the spatial inhomogeneity within the photoactive layer. This has to be taken into consideration and therefore for fitting purpose the capacitor element is replaced by constant phase elements (CPE).^39^ Impedance for an ideal capacitor and CPE is given by eqn (1) and (2) respectively.^40^ From above equations, it is apparent that CPE is equivalent to capacitance if *P* equals 1.1
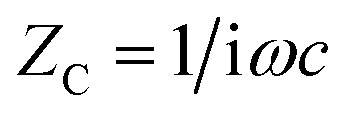
2
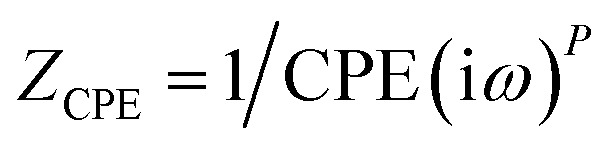
3
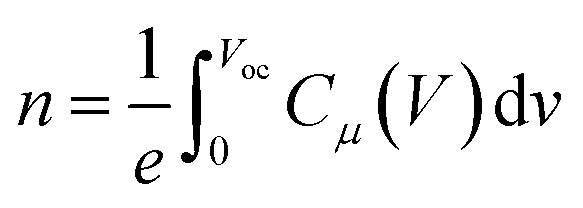


After fitting, values of recombination resistance and chemical capacitance is obtained and is further used to calculate the value of carrier density using eqn (3). Response time representative of the recombination processes is calculated from the characteristic frequency (*ω*) at the top of the arc, where 2π*ω* = 1/*τ*.^40,41^ It is observed that for both the system recombination lifetime lies in order of 10^−6^ s. Further, as can be seen in Fig. 4(c), recombination resistance is less in case of best performing device (Cu_2_S NCs, 3 wt%) when compared to reference device (Cu_2_S NCs, 0 wt%). Additionally, using eqn (3), we have calculated the charger carrier density (*n*) for both the systems using the capacitance values shown in Fig. 4(d). It is found that carrier densities for best performing device (Cu_2_S NCs, 3 wt%) and reference device (Cu_2_S NCs, 0 wt%) at 0.8 V is ∼5 × 10^17^ cm^−3^ and ∼3 × 10^17^ cm^−3^ respectively. Higher charge carrier density for the best performing device (Cu_2_S NCs, 3 wt%) suggest that the charge collection is more efficient in this system. Thus, it can be said that the presence of Cu_2_S NCs helps in improving the charge transfer and charge collection by providing electron cascade. This increase in the *J*_sc_ and FF and therefore the PCE supports the theory of charge transfer complex (CTC)^37^ wherein the formation of the electron cascade structure of the well-placed conduction band of the Cu_2_S NCs between LUMO of donor polymer PTB7-Th and PCBM polymer plays a vital role. Also, it might be possible that the presence of Cu_2_S NCs in the photoactive blend provides additional energetically favorable interfaces necessary for the exciton diffusion and formation of CT states.

**Fig. 5:**
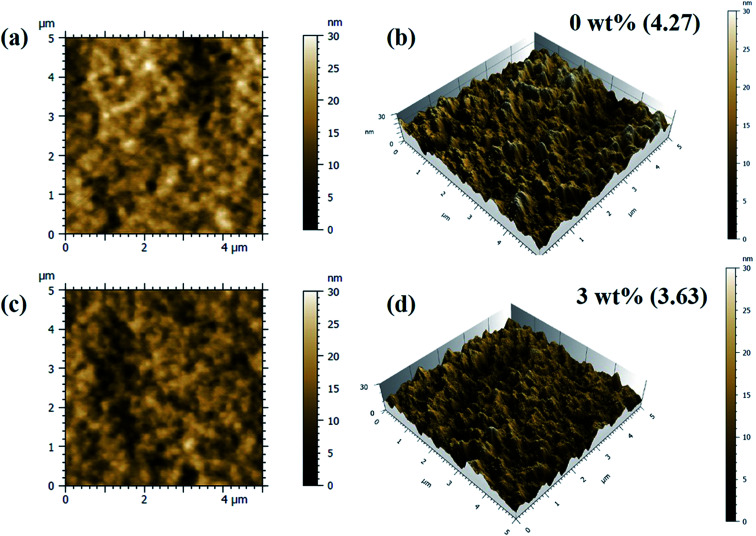
2D and 3D AFM images of the films of PTB7-Th:Cu_2_S NCs:PC_70_BM photoactive blend with (a and b) 0 wt% (c and d) 3 wt% of Cu_2_S NCs.

AFM studies of the spin casted films of binary and ternary photoactive blends was carried out and 2D and 3D images are shown in the Fig. 5 and S2.† As can be seen, films are quite smooth and does not have any pin-holes. The rms values of surface roughness are found to decrease from ∼4.27 nm for the films with 0 wt% of Cu_2_S NCs to ∼3.63 nm for the films with 3 wt% of Cu_2_S NCs. However, with the further increase in the concentration of Cu_2_S NCs, rms surface roughness increases to 3.77 nm. This might be due to the possibility of inhomogeneous distribution/agglomeration of Cu_2_S NCs in the matrix. Accordingly, this has decreased the additional energetically favorable interfaces thereby affecting the process of exciton diffusion and CT states formation. The shielding effect could be due to the higher concentration of NCs which inherently affects the transport of electron in the matrix. This might have increased the charge carrier recombination which ultimately reduces the PCE.

## 4. Conclusion

In conclusion, role of Cu_2_S NCs as third component in ternary OSCs has been successfully investigated by varying the concentration of Cu_2_S NCs in ternary photoactive blend comprised of PTB7-Th, PCBM and Cu_2_S NCs. Fabricated ternary OSCs having a device architecture ITO/ZnO/PTB7-Th:Cu_2_S NCs:PCBM/MoO_3_/Ag has shown the improved PCE of 8.20% as against the PCE of 6.96% for the reference device. The improvement in the performance parameters has been explained on the basis of the EIS measurements and AFM studies which suggest that presence of Cu_2_S NCs facilitates formation of cascading energy levels and thus helps in suppressing trap-assisted recombination.

## Conflicts of interest

Authors declare no conflict of interest.

## Supplementary Material

RA-009-C8RA08919A-s001
