# Supplementary material for: Enhanced performance of PTB7-Th:PCBM based active layers in ternary organic solar cells
Source: RSC Adv. 2019 Mar 6;9(13):7457–63. doi: 10.1039/c8ra08919a (PMC9061204; doi:10.1039/c8ra08919a)
Supplement: RA-009-C8RA08919A-s001 [file RA-009-C8RA08919A-s001.pdf]

Electronic Supplementary Material (ESI) for RSC Advances.
This journal is © The Royal Society of Chemistry 2019

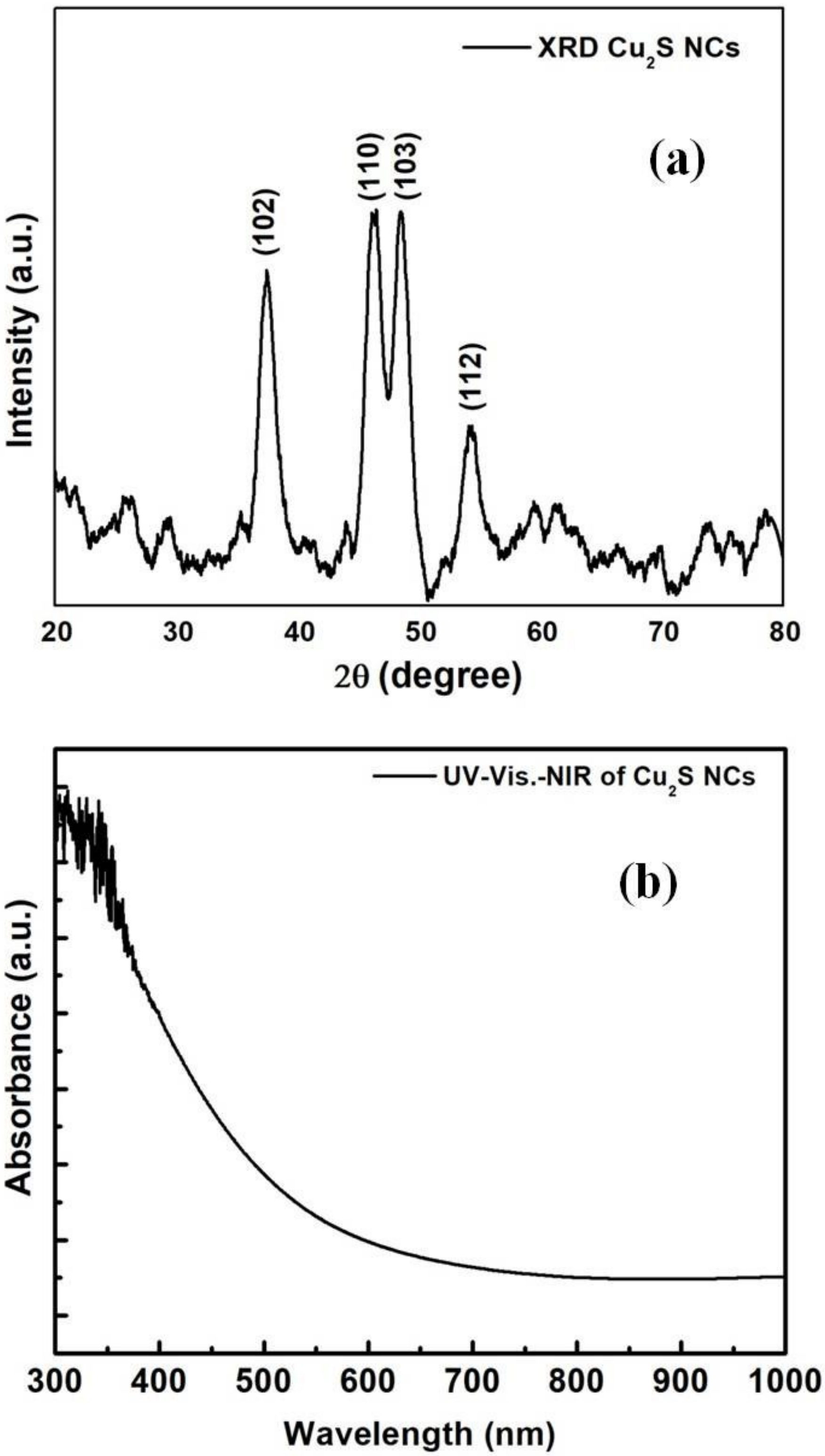

Fig. S1 (a) XRD pattern of as synthesized Cu_2S NCs (d) UV- Vis. NIR absorption spectra of as synthesized Cu_2S NCs.

The crystalline quality has also been confirmed by X-ray diffraction (Cu K_{α} radiation, λ = 1.5418 A^{o}) of Cu_2S NCs, as shown in the Fig. S1 (a) with 2θ scan between 20° to 80°. Presence of all the prominent peaks and their corresponding 2θ values are in close agreement with the previous studies and replicates with the JCPDS file no.00-026-1116. This confirms the hexagonal structure of as-synthesized Cu_2S NCs. Absence of additional peaks besides the peaks of Cu_2S NCs confirms that the adopted protocol yields the pure phase of Cu_2S NCs which is essential for the device fabrication. The advantage of using Cu_2S nanocrystals is having low band-gap as shown in Fig. S1 (b) in absorbance spectra UV-Vis.-NIR, where wide absorption spectrum can be seen up of wavelengths below 1000 nm and therefore capable of harnessing the major part of the solar spectrum.
